# Chest pain and high troponin level without significant respiratory symptoms in young patients with COVID-19

**DOI:** 10.22088/cjim.11.0.561

**Published:** 2020

**Authors:** Behnam Hedayat, Kaveh Hosseini

**Affiliations:** 1 Department of Cardiology, Tehran Heart Center, Tehran University of Medical Sciences, Tehran, Iran

**Keywords:** COVID-19, acute myocarditis, acute coronary syndrome

## Abstract

**Background:**

Of all patients infected with COVID-19, 95% have mild symptoms, but 5% may experience severe illness. There are reports of myocardial injury associated with the COVID19 infection in middle-aged and old people with baseline cardiac conditions. Acute myocardial injury has been suggested as a marker for disease severity. Sometimes it is hard to differentiate between acute coronary syndrome and acute myocarditis; hence detailed history taking, lab tests and imaging will be necessary.

**Case Presentation:**

Herein, we described two young patients presenting with chest pain and no significant respiratory symptoms, one without cardiovascular risk factors and another one with diabetes mellitus and cigarette smoking. COVID-19 was documented with real-time reverse-transcriptase-polymerase chain reaction (rRT-PCR).

**Conclusion:**

Early Chest CT scan besides coronary CT angiogram (if available) in suspicious cases can help physician to make fast decisions. These two cases both had complication-free hospital stay. Despite markedly high on-admission troponin levels, which is known as a marker of poor prognosis they discharged in good condition. One month follow-up was also uneventful.

In December 2019, the Coronavirus emerged in the Huanan Seafood Market and rapidly became a world-wide problem (1). On February 11, 2020, it was termed “COVID-19”. Now a pandemic, COVID-19 is considered a real crisis. Fortunately, of all patients infected with COVID-19, 95% have mild symptoms (3), but 5% may experience severe illness. The latest reports indicate that older patients with cardiovascular disease constitute the highest-risk group. There are reports of myocardial injury associated with COVID19 infection, and it is recognized as a sign of increased risk of death in such patients (4, 5). Herein, we present two cases of young adults with diagnosis of COVID19 associated with myocardial injury but without significant respiratory symptoms at presentation. Subtle on-admission Electrocardiogram (ECG) changes were recorded in both cases. 

## Case presentation

**Patient 1:** was an otherwise healthy 24 years old man presented with chief complaint of retrosternal chest pain in the past 5 days prior to the presentation. He did not have conventional risk factors for premature coronary artery disease. He described the pain radiates to his left arm and occurred in an intermittent fashion not related to exertion; each episode lasts about 2 hours. The pattern of chest pain was atypical. He mentioned sore throat and cold sweating without coughing. In the emergency room, his blood pressure was 120/60 mmHg and heart rate was 115 bpm. Oxygen saturation by pulse oximetry was 94% while breathing ambient air and tympanic temperature was 36.7°C.

**Figure 1:**
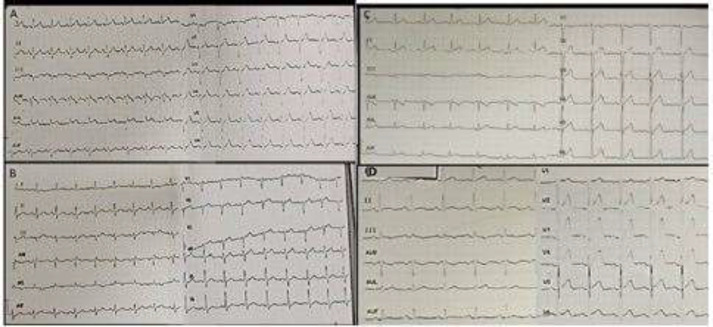
Electrocardiography of patients. A,B: ECG of the patient 1 on the day of admission and second day of admission, respectively. C,D: ECG of patient 2 on the day of admission and 3rd day of admission, respectively, showing slight ST segments coving in inferior leads, most prominent in 3rd day

Electrocardiography showed sinus tachycardia, mild ST segment elevation less than 1 mm in lead I and aVL and ST segment depression of about 1 mm in leads III and aVF (figure 1, panel A&B). First cardiac hs-TroponinT result was 911 ng/L (cut off <24ng/L). He was admitted with diagnosis of non ST elevation MI (NSTEMI) or myocarditis. Results of 1 hour cardiac Troponin T was 1155 ng/L. Other Baseline laboratory tests were presented in table1.

**Table1:** Laboratory tests results

	**Patient 1**		**Patient 2**	
**Laboratory Findings**	**day of admission**	**day of discharge**	**day of admission**	**day of discharge**
White cell count (per mm^3^)	10,510	9,700	9,000	9,800
Differential count (per mm^3^)				
Total neutrophil	7,451	5,907	5,859	4,645
Total lymphocyte	2,610	3,104	2,745	4,517
Platelet count (per mm^3^)	205,000	325,000	152,000	222,000
Hemoglobin (g/dl)	16.1	16.5	14.7	15.9
Urea(mg/dl)	24	45.6	25.2	29.2
Creatinine (mg/dl)	1.01	1	1.06	0.96
EGFR*(ml/min/1.73m2)	96.5	97.6	88.4	99.1
Aspartate aminotransferase (IU/L)	48			
Alanine aminotransferase(IU/L)	21			
LDH (IU/L)	677			
Low density lipoprotein(LDL) (mg/dL)	66		86	
Triglyceride (mg/dL)	95		257	
Brain natriuretic peptide(ng/L)		<15		
CRP (Nl <0.5 mg/dl)	11.17	0.66	4.08	1.67
Troponin T (time zero) (ng/L)	911.1		123	
Troponin T (1hr) (ng/L)	1155		113	
Procalcitonin(ng/ml)			0.2	
D-Dimer			0.29	

* EGFR denotes estimated GFR and calculated with MDRD method

**Figure 2:**
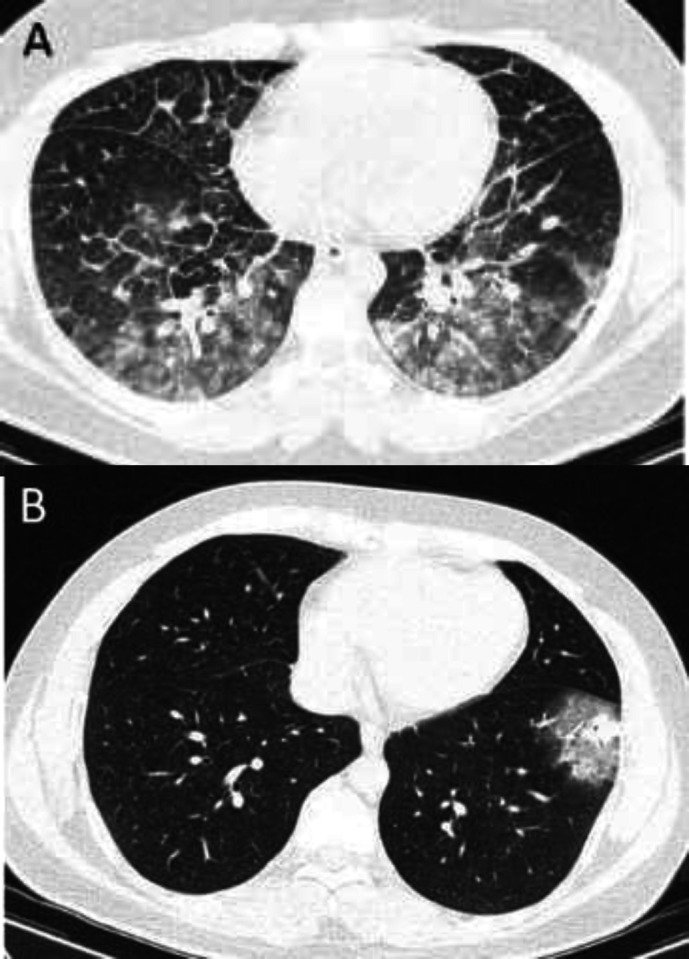
Lung CT scan of both patients. A, lung CT of patient 1 showed bilateral peripheral ground glass opacities in lower lobes, compatible with COVID19. B, lung CT of patient 2 revealed subpleural ground glass density in lower lobe compatible with COVID-19

**Figure 3:**
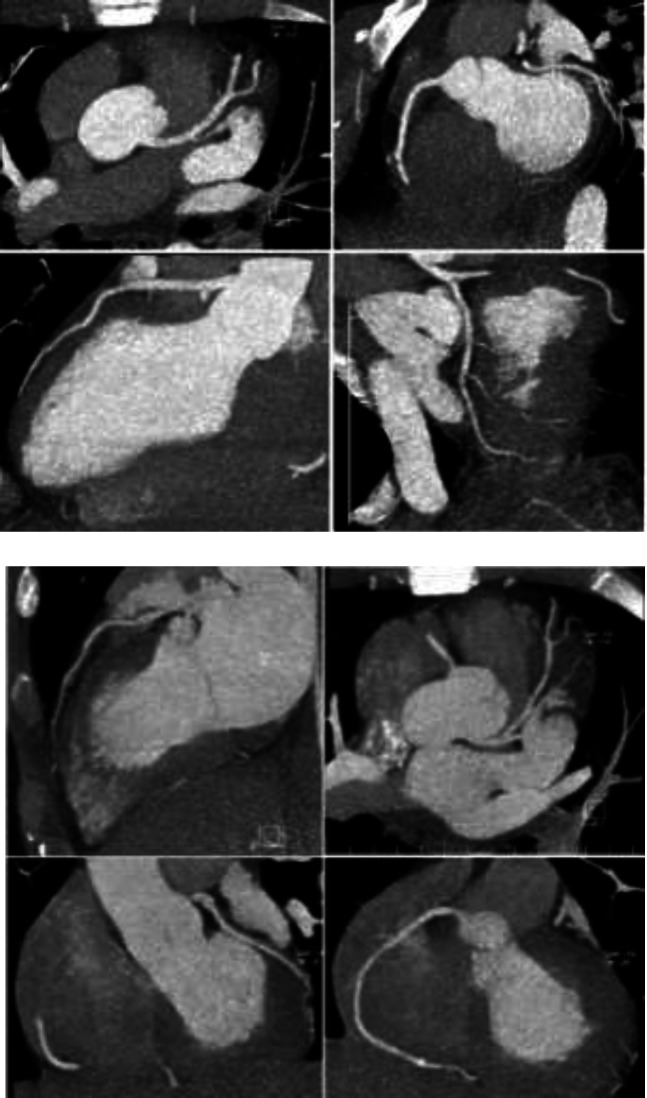
Coronary CT angiography of patient 1 (upper panel four images) and patient 2 (lower panel four images) showing normal appearing coronary arteries

Because of symptoms of sore throat, sweating and also elevated CRP, nasopharyngeal sample was taken and sent for SARS-COV-2 RT-PCR. Lung computed tomography scan revealed bilateral ground glass peripheral densities in lower lobes which was compatible with CT findings of COVID-19 (figure 2, panel A). Echocardiography showed normal LV size and systolic function with ejection fraction of about 55%. There was neither regional wall motion abnormality nor pericardial effusion. Results of RT-PCR test became positive which was in concert with lung CT scan findings. Lopinavir/ritonavir was initiated. Coronary multi-detector CT scan was performed and revealed normal coronary arteries (figure 3, panel A). Based on findings mentioned, final diagnosis of myocarditis due to COVID19 was made. On the second day, ECG showed resolution of ST deviations and appearance of T wave inversion in lead I and aVL. Hospital course was uneventful. He was discharged after 6 days of admission and improvement of symptoms, with recommendation of self-isolation for at least 14 days. Metoral 25 mg bid was prescribed. In 1 week telephone follow up, he was in good clinical condition and his symptoms had been improved substantially. 

**Patient 2:** was a 28 years old man presented with retrosternal chest pain which had occurred at 4:00 AM, 12 hours before presentation. He described his pain both exertional and positional. The pain was not relieved by sublingual nitroglycerine. He had history of diabetes mellitus and was current a cigarette smoker and a morphine user. He also reported episodes of fever, chill, fatigue, rhinorrhea and headache in four days prior to presentation. No coughing episodes were reported in his history. Chest CT had been performed in an outpatient clinic, and as he explained it was said to be normal, but he did not provide any documented image or report. 

In emergency department he was alert and oriented, his blood pressure was 125/70 mmHg, heart rate was 89 bpm, respiratory rate was 18 per minute, O2 saturation by pulse oximetry was 98% while breathing room air, and tympanic temperature was 36.8 °C. Electrocardiography was normal except for slight coving of inferior leads ST segments (figure 2, panel B).

Baseline and 1 hour cardiac hs-troponin T levels elevated; 126 ng/L and 113ng/L respectively. Other laboratory tests were shown in table 1. Echocardiography revealed normal LV size with mild systolic dysfunction and ejection fraction of about 45% with global hypokinesia. Lung CT scan was performed and revealed ground glass sub-pleural density in left lower lobe which was compatible with COVID19 diagnosis. Hydroxychloroquine was initiated with first dose of 400 mg twice daily then 200mg twice daily. Nasopharyngeal swab sample was sent and result became positive for SARS-COV-2. 

He underwent coronary CT angiography which showed normal appearing coronary arteries (figure 3, panel B). Myocarditis with COVID19 was diagnosed. Hopefully, hospitalization period was without complication. Electrocardiography on the 3^rd^ day of admission showed the same ST coving but slightly more prominent than before. After 4 days of admission, he was discharged and recommended to be home quarantined. Metoprolol succinate 47.5 daily and Enalapril 2.5 daily was prescribed. Treatment with hydroxychloroquine was continued for total 10 days after COVID19 diagnosis. He was doing well in after 1 week telephone follow-up. 

## Discussion

Severe acute respiratory syndrome coronavirus 2 (SARS-COV-2) which is nominated as novel coronavirus disease (COVID 2019) by World Health Organization (WHO), has affected more than 6 million individuals around the world and was the main cause of more than 370,000 deaths till the first of June 2020 according to the WHO report (6). 

On March 11, 2020, WHO declared the coronavirus outbreak a pandemic and involved various countries around the world (7-10). Iran, the second largest country in the Middle East also officially announced the first case of the disease in Qom province in February, 2020. Based on the current report, the disease spread to most provinces of the country. There are several factors associated with poor outcomes in these patients including old age, comorbidities, cardiovascular disease, renal dysfunction, etc. (11). According to the available studies, different patients with COVID-19 have been evaluated descriptively and in some investigations prognostic factors have been identified (12-14). Herein, we described two young patients with chest pain and no significant respiratory symptom along with normal on-admission O2 saturation, one without cardiovascular risk factors and another one with diabetes mellitus and cigarette smoking. 

Electrocardiography, type of chest pain in detailed history and baseline hs-troponin level was in favor of acute coronary syndrome, especially in the second patient with two conventional coronary risk factors. However, young age and absence of significant rise and fall pattern in hs-troponin should be an alarming sign for probable myocarditis. Early chest CT scan besides coronary CT angiogram (if available) in suspicious cases can help physician to make fast decisions. Young patients with COVID-19 may not have significant coughing or low O2 saturation at presentation and may present with chest pain, malaise, rhinorrhea and high troponin in lab tests. High troponin level is not necessarily associated with worse outcome and many other factors such as young age play an important role in disease course.

 Cardiac magnetic resonance is an accurate tool to differentiate acute myocarditis and acute coronary syndrome; however, the exam time, high cost and availability are main limiting factors. Fortunately, these two cases both had uneventful and complication-free hospital courses and were in good clinical status at follow up, despite markedly elevated troponin levels at the time of admission, which is known as a marker of poor prognosis and higher risk of death in COVID-19. 
